# Predictors of an Initial Academic Position in Emergency Medicine

**DOI:** 10.5811/westjem.2018.10.39096

**Published:** 2018-11-13

**Authors:** Terry Singhapricha, Olivia Minkhorst, Timothy Moran, Jonathan Swanson, Philip Shayne

**Affiliations:** * Emory University, Department of Emergency Medicine, Division of Ultrasound, Atlanta, Georgia; † Emory University, Department of Emergency Medicine, Atlanta, Georgia

## Abstract

**Introduction:**

Each year, emergency medicine (EM) residency graduates enter a variety of community and academic positions. For some training programs, the potential for an academic career is a consideration during the interview process; however, no studies have looked at factors that might predict an academic career. Our goal was to identify variables present during the EM application cycle that predict an initial academic position.

**Methods:**

We retrospectively reviewed application materials from 211 EM graduates at Emory University from 2003–2013. We analyzed biographical variables, board scores, personal statements, and both undergraduate and medical school research experience and publications. An academic position was defined as working at a site with residents rotating in the emergency department, full or part-time appointment at a medical school, or a position with research required for promotion. We used a logistic regression model to determine the impact of these predictors on obtaining an initial academic position.

**Results:**

A total of 79 (37%) graduates initially chose an academic job, and 132 (63%) took a community position. We identified the following statistically significant variables: younger age (odds ratio [OR] [0.79], 95% confidence interval [CI] [0.67–0.93], p=0.01); undergraduate publications (OR [1.41], 95% CI [1.08–1.83], p=0.01); and medical school publications (OR [3.39], 95% CI [1.66–6.94], p<0.001). Of note, mention of an academic career in the personal statement showed no statistical correlation (p = 0.41).

**Conclusion:**

Younger age, and undergraduate and medical school publications were the variables most associated with an initial academic position. As this is a single-institution study, more studies are needed to validate these findings.

## INTRODUCTION

Each year approximately 2,000 emergency medicine (EM) residents will graduate and obtain positions at academic, community, or fellowship sites.^1^ Of these graduates, approximately 39.6% will take either an academic or fellowship position, while 57.1% will take a community position and the remainder will pursue other careers.^2^ The only related EM-based study, done by Burkhart et al. in 2011, found that larger programs, location, and resident academic productivity may lead to more graduates choosing an academic career.^2^ This study received survey responses from 103 of 147 residency programs and found a greater proclivity for a career in academic medicine from EM programs with more than eight residents as well as programs located in the Northeast/Mid-Atlantic and Southwest regions.

To date, there have been no studies that have examined whether any factors on an individual’s Electronic Residency Application Service (ERAS®) file might predict an initial position in academic medicine. Studies from a variety of other specialties suggest that factors such as female gender, undergraduate and medical school research, United Stated Medical Licensing Examination (USMLE) scores, and peer-reviewed, pre-residency publications may be associated with a higher likelihood of an academic career.^3^–^8^ These results, however, are inconsistent, and likely the factors that influence an individual’s career choice may be distinct for each specialty.

Although EM residency programs were initially more clinically oriented with only 30% of programs in 1994 affiliated with an academic center,^9^ there has been a general push to grow EM as an academic specialty that balances clinical care with research and education.^10^ For many programs, an applicant’s potential career choice plays a factor in recruitment, with different programs seeking to train varying numbers of academic clinicians; however, there are no studies in the field of EM that have looked at which factors on a candidate’s application would predict an academic position. Based on studies from other fields, our hypothesis was that applicants who published more papers in undergraduate and medical schools would more likely choose to initially pursue a position as an academic clinician. Furthermore, we sought to seek any other variables present in residency applications that are associated with taking an initial position in academics, and we looked at variables known to be associated with an academic career based on previously published studies, such as attendance at more research-oriented medical schools and an applicant’s USMLE step score.^3^–^5^

Our overall objective was to determine if any factors present on an EM residency application would be a predictor of an initial career in academics. Our goal is to use this data in the future, along with the interview process, to help with resident recruitment as well as help shape resident professional development during training.

## METHODS

This was a retrospective, single-institution study of the application materials of residents who graduated between 2003 and 2013 from the Department of Emergency Medicine at Emory University. Residents were grouped for analysis with their entering class, and we excluded from the study those who did not complete training at the program. All graduates of the program during this time period were included in the study. Biographical, undergraduate, and medical school data were recorded from residency application materials of these classes archived onsite. This study was deemed exempt by the institutional review board. For the purposes of this study we defined an academic setting as a site that require supervision of residents in the emergency department (ED), full-or part-time appointment at a medical school, or a position with academic research required for promotion. This definition is similar to previous studies^2^ and was chosen to broadly include all physicians who would define themselves as academic clinicians.

**Population Health Research Capsule**

What do we already know about this issue?

*In the field of EM, there are no studies investigating what factors on residency applications would lead to graduates taking initial academic jobs*.

What was the research question?

Is research experience prior to residency a predictor of initial academic jobs after graduation?

What was the major finding of the study?

*Younger age and publications prior to residency are independent predictors of an initial academic position*.

How does this improve population health?

*Analysis of residents’ career choices could allow for improved mentorship and selection that will better reflect each program’s mission and the communities they serve*.

Factors were chosen based on findings in other fields associated with a likelihood of an academic career as listed in Table 1. Biographical data included gender, age at the time of application, other advanced degrees in addition to medical doctor, or a prior career and were extracted directly from archived application forms. Other advanced degrees include a master’s degree, or PhD, and did not have to be from a scientific field. Prior career was defined as any previous position lasting longer than one year that was not designed to get clinical or research experience for a career in medicine, or any job position that lasted for longer than five years.

We extracted undergraduate research experience from self-reported data on each archived application, and then confirmed publications via searches on PubMed and Google Scholar. Manuscripts published after graduation from research done while an undergraduate were still marked as an undergraduate publication. Research and manuscripts done after undergraduate graduation but before medical school were also counted in the undergraduate category.

Medical school data included research experience, manuscript publications, membership in Alpha Omega Alpha (AOA) honor society, USMLE Step 1 and Step 2 scores, a personal statement with references to a career in academics, and attendance at a “research-heavy” medical school, classified as appearing in the top 10 list of “Research Medical Schools” in the *US News and World Report* rankings during the years of the study.^11^ This list of research-heavy schools during this time period is shown in Table 2. A personal statement considered positive for a reference to an academic career was one in which the resident applicant specifically stated that he or she wished to pursue a career in academic medicine or at an academic institution. During data collection, we resolved any discrepancies with scoring the subjective variable “personal statement mentioning academics” after consensus discussion.

We classified residency graduates into academic and non-academic categories depending on their initial job following residency or fellowship graduation. An academic position was defined as a job in which the graduate would directly work with and supervise residents rotating through the ED affiliated with an accredited graduate medical education program. Of note, this did not include only EM residents. This definition was chosen to be consistent to be inclusive for all physicians who would consider themselves an academic clinician and an educator even though they may not be employed directly by a medical school or university. The location of where the graduates initially practiced was acquired from their residency archive file, which is continuously updated by our administrative staff.

We used a binary logistic regression to evaluate the importance of each predictor. Statistical analyses were conducted using SPSS (v. 24; Armonk, New York).

## RESULTS

We included a total of 211 EM graduates in the analyses. As shown in Table 3, 79 graduates (37%) chose an initial position in academics while 132 (63%) initially took a community position. As shown in Table 4, the statistically significant factors associated with an academic position were younger age, undergraduate publications, and medical school publications. In terms of age, the correlation was inversely related to each increasing year of age (odds ratio [OR] [0.79] per year, 95% confidence interval [CI] [0.67–0.93], p=0.01). Other factors, such as gender, previous career, advanced degrees, conducting research in undergraduate and medical school, a research-heavy medical school, USMLE scores, and AOA membership, showed no statistical association with an initial academic position. Furthermore, mention of pursuing a career in academics in a personal statement was not associated with an initial academic position (p=0.41).

## DISCUSSION

This study sought to determine the factors that would predict an initial position in academic EM. To our knowledge, this is the first study in EM that looks at individual factors associated with academics, and the correlation of an applicant’s undergraduate publications and medical school publications is consistent with findings reported in other fields.^3^–^5^,^7^ A unique finding is an inverse relationship with increasing age and a position in academics, which has not been shown in studies from other fields.^13^–^14^ It is unclear why age is a specific predictor in this study compared to other fields as most academic tenure tracks have similar promotion processes; however, a potential reason may be that EM is unique in that shift-work allows for more schedule flexibility, and older physicians who would potentially have more personal responsibilities such as families would prefer the increased flexibility, and potentially greater pay at community sites.

The correlation of publishing with an initial academic position was something hypothesized to be a predictor as it has been consistent with studies in other fields. The act of publishing in a peer-reviewed journal shows a level of interest and dedication to conducting research, especially in EM as it is not as research intensive as other specialties. We suspected that individuals arriving into residency with an interest in research would likely take an initial academic position where there are potentially more research opportunities and protected time than with a community position.

Other factors such as gender, a prior career, undergraduate, or medical school research experience showed no significant correlation to an academic position. Unlike studies in other fields, neither an advanced degree, a research-intensive medical school, nor USMLE scores predicted an academic position.^4^,^6^,^7^ The lack of an association with USMLE scores and AOA membership with respect to academics is more likely specific to the field of EM, which usually evaluates these factors in the context of a whole application as opposed to a threshold or screening criteria. Furthermore, the lack of an association between an academic career and *US News and World Report* rankings of a research-heavy medical school is likely attributable to EM’s greater emphasis on clinical and population-based research, which is not as well reflected as basic science in these rankings.

Although 39 (18.5%) of the graduates stated in their personal statement that they wished to pursue an academic career, only 17 of those graduates took an initial job in academics, which demonstrated no statistical significance. The reasons behind this discrepancy are beyond the scope of our study but could potentially be the result of changing life priorities as a resident goes through training, and the colloquial belief that when applying at academic centers they must express a desire to pursue academics to be considered for a spot in the program.

## LIMITATIONS

The major limitations of this study are those associated with a single-institutional study and whether the results are generalizable to other programs. The rates of residents who pursue an initial academic, fellowship, or community career are similar to rates previously described in the literature.^2^ The finding that pre-residency publications are associated with an academic career is consistent with findings in a variety of other fields. In addition, the review was retrospective and historical; graduating residents’ career decisions might change with the clinical landscape. Furthermore, the use of *US News and World Report* rankings as a measure of research productivity is controversial; however, this method has been used by previous authors and the methodology is well described.^4^,^12^ Lastly, although our study was similarly powered to studies in other fields, the presence of 13 variables and 79 total applicants who went into academics lends itself to the risk of overfitting, and a large-scale multi-institutional study would be beneficial to validate these findings. It is also important to note that this study looks at an initial position in academic medicine; however, many individuals may spend a year or more at an academic institution while they are deciding on a career choice. This may lead to an overestimation of how many graduates ultimately pursue a career in academics.

## CONCLUSION

In summary, this was a pilot study that looked for individual residency-application factors that would predict a graduate’s initial career choice to pursue academic EM. Among the 13 variables analyzed over this 10-year period, we found an association between an initial academic position and having peer-reviewed publications during undergraduate and medical school, as well as with younger age. The act of publishing itself was statistically significant, but research experience itself was unrelated. Other EM studies are needed to validate these results.

## Figures and Tables

**Table 1:** Variables studied.

Age
Gender
Previous career (yes/no)
Advanced degree (yes/no)
Undergraduate research (yes/no)
Number of undergraduate publications
Research heavy medical school (yes/no)
Medical school research (yes/no)
Number of medical school publications
Personal statement mentioning academics
USMLE step 1 score
USMLE step 2 score
AOA membership

*USMLE*, United States Medical Licensing Exam; *AOA*, Alpha Omega Alpha.

**Table 2:** *US News and World Report’s* top 10 research medical schools (2000–2010).

Columbia University
Duke University
Harvard University
Johns Hopkins University
Stanford University
University of California at Los Angeles
University of California at San Francisco
University of Chicago
University of Michigan
University of Pennsylvania
University of Washington
Washington University in St. Louis
Yale University

Please note that there are a total of 13 schools that ranked in the top 10 research schools during this time period.

**Table 3:** Baseline demographics of graduates of Emory University’s emergency medicine residency program.

Characteristic	% or M	95% CI or IQR
Female (%)	46.92	40.19; 53.65
Age (M)	27	3
Advanced degree (%)	17.54	12.4; 22.67
Prior career (%)	15.64	10.74; 20.54
Undergraduate research (%)	69.19	62.96; 75.42
Medical school research (%)	63.51	57.01; 70.0
Undergraduate publications (%)		
0	76.3	70.57; 82.04
1	10.9	6.7; 15.11
2	8.53	4.76; 12.3
3	2.37	0.32; 4.42
4	0.95	0.0; 2.26
7	0.47	0.0; 1.4
17	0.47	0.0; 1.4
Medical school publications (%)		
0	82.46	77.33; 87.6
1	12.32	7.89; 16.76
2	3.79	1.21; 6.37
3	0.47	0.0; 1.4
4	0.47	0.0; 1.4
6	0.47	0.0; 1.4
USMLE 1 (M)	222	24.6
USMLE 2 (M)	230.03	26.1
Academic personal statement (%)	16.78	11.74; 21.82
AOA (%)	10.43	6.3; 14.55
Academic career (%)	37.44	30.91; 43.97

*USMLE*, United States Medical Licensing Exam; *AOA*, Alpha Omega Alpha; *M*, median value; *CI*, confidence interval; *IQR*, interquartile range.

**Table 4:** Predictors of an initial academic position.

Predictor	Odds ratio	95% CI	P value
Gender	1.42	0.72; 2.78	0.31
Age	0.79	0.67; 0.93	0.01
Prior career	2.05	0.65; 6.41	0.22
Undergraduate research	1.57	0.74; 3.33	0.24
Undergraduate publications	1.41	1.08; 1.83	0.01
Advanced degree	1.76	0.69; 4.52	0.24
Research focused medical school	0.38	0.08; 1.88	0.23
Medical school research	0.71	0.35; 1.43	0.34
Medical school publications	3.39	1.66; 6.94	<.001
Academic personal statement	1.43	0.61; 3.35	0.41
USMLE 1	0.99	0.96; 1.02	0.55
USMLE 2	1.01	0.98; 1.04	0.65
AOA	0.78	0.25; 2.45	0.67

*USMLE*, United States Medical Licensing Exam; *AOA*, Alpha Omega Alpha; *CI*, confidence interval.

P values refer to the results of binary logistic regression analysis for academics versus non-academics.
